# Macroalgae Derived Fungi Have High Abilities to Degrade Algal Polymers

**DOI:** 10.3390/microorganisms8010052

**Published:** 2019-12-26

**Authors:** Aleksandrina Patyshakuliyeva, Daniel L. Falkoski, Ad Wiebenga, Klaas Timmermans, Ronald P. de Vries

**Affiliations:** 1 Fungal Physiology, Westerdijk Fungal Biodiversity Institute & Fungal Molecular Physiology, Utrecht University, Uppsalalaan 8, 3584 CT Utrecht, The Netherlands; a.patyshakuliyeva@nioo.knaw.nl (A.P.); dalf@novozymes.com (D.L.F.); a.wiebenga@westerdijkinstitute.nl (A.W.); 2 NIOZ Royal Netherlands Institute for Sea Research, Landsdiep 4, 1797 SZ ′t Horntje, The Netherlands; klaas.timmermans@nioz.nl

**Keywords:** seaweed biomass, enzymes, algal polysaccharides, marine fungi

## Abstract

Marine fungi associated with macroalgae are an ecologically important group that have a strong potential for industrial applications. In this study, twenty-two marine fungi isolated from the brown seaweed *Fucus* sp. were examined for their abilities to produce algal and plant biomass degrading enzymes. Growth of these isolates on brown and green algal biomass revealed a good growth, but no preference for any specific algae. Based on the analysis of enzymatic activities, macroalgae derived fungi were able to produce algae specific and (hemi-)cellulose degrading enzymes both on algal and plant biomass. However, the production of algae specific activities was lower than the production of cellulases and xylanases. These data revealed the presence of different enzymatic approaches for the degradation of algal biomass by macroalgae derived fungi. In addition, the results of the present study indicate our poor understanding of the enzymes involved in algal biomass degradation and the mechanisms of algal carbon source utilization by marine derived fungi.

## 1. Introduction

Macroalgae represent a promising renewable resource for the production of sustainable bioenergy and biomaterials and as ingredients in the development of new food and feed products [1]. They are fast growing and rich in proteins, carbohydrates, bioactive compounds, vitamins, and macro- and microelements. Interest in macroalgae-based research has recently increased due to the rise in the world population and expected greater demand for food, feed, and fuel. One of the main challenges to efficiently extract valuable compounds from macroalgal biomass is structurally rigid and complex cell wall polysaccharides of seaweeds [2,3,4]. Cell wall polysaccharides of seaweeds are species dependent and significantly different from terrestrial plants. For instance, they include ulvan, a sulfated glucuronoxylorhamnan, in green macroalgae *Ulva lactuca* [5]; alginate and sulfated polysaccharides, such as fucoidan, in brown macroalgae, such as *Saccharina latissima*, *Laminaria digitata,* and *Fucus vesiculosus* [4,6,7]. Macromolecular composition of seaweed is also varied with seasons and nutrient availability [8,9]. For instance, it has been shown that the highest carbohydrate content was in spring [10] and summer [11] in green seaweed, *U*. *lactuca*, with increased rhamnosyl and glucuronoyl residues in April [10]. In contrast, in brown seaweed, *S*. *latissima*, it was demonstrated that alginate content was the highest during summer and the lowest during autumn [12,13]. In addition to algae-specific polysaccharides, seaweeds contain cellulose which is also a structural component of plants. However, cellulose in most plants is complexed with lignin, while this is not the case in macroalgae, enabling easier degradation or extraction. The presence of lignin in plant cell wall and its association with cellulose hinders the accessibility of plant cell wall polysaccharides to enzymatic treatment such as decreases in the length of cellulose that can be processed or limits the surface area for enzymes to bind [14,15,16].

Current methods of breaking up the macroalgal cell wall to extract valuable compounds are not optimal and have disadvantages of high energy consumption in case of mechanical methods, harsh conditions and toxicity during chemical extraction, and generation of side products and waste if high temperatures are used [17,18,19]. In contrast, it has been demonstrated that enzyme-assisted extraction holds big potential for development of targeted and sustainable processing of seaweeds [2,3,20,21]. Therefore, identification of enzymes involved in the specific degradation of algal cell walls is very important.

Fungi represent a large and diverse group of microorganisms in microbiological communities in the marine environment and have an important role in nutrient cycling [22]. They are divided into two major groups; obligate marine fungi and facultative marine fungi [23]. Obligate marine fungi are adapted to reproduce in the aquatic environment, while facultative marine fungi can grow in aquatic as well as terrestrial environments [23]. Marine fungi are called marine-derived fungi when their facultative or obligate state is not certain [23]. Marine fungal species occur as saprobes, parasites, or symbionts and colonize a wide range of substrates, such as sponges, corals, mangroves, seagrasses and algae [24,25]. Marine fungi associated with algae are largely unexplored, despite their ecological role and potential industrial applications. For example, it has been shown that fungi associated with algae produce many bioactive secondary metabolites [26,27,28]. Algae derived fungi can be associated with a variety of algae, including brown (e.g., *Agarum clathratum*, *Fucus* sp., *Laminaria* sp., *Sargassum* sp.), green (e.g., *Ulva* sp., *Enteromorpha* sp., *Flabellia* sp.), or red (e.g. *Chondrus* sp., *Dilsea* sp., *Ceramium* sp.) algae [29,30,31,32,33].

The most commonly described fungi associated with algae belong to the Ascomycota and are represented by a wide diversity of genera such as *Acremonium*, *Alternaria*, *Aspergillus*, *Cladosporium, Phoma*, *Penicillium*, *Trichoderma*, *Emericellopsis*, *Retrosium*, *Spathulospora*, *Pontogenia,* and *Sigmoidea* [30,31,32,34,35,36]. Many of these genera also contain terrestrial species that are known to produce plant biomass degrading enzymes that convert lignocellulosic material into different high value products including biofuels and biochemicals [37]. For instance, enzymes from terrestrial species of *Trichoderma* [38] and *Aspergillus* [39] have been produced commercially for industrial applications. However, production of plant based biofuels and other high value products have some disadvantages, which include competition with agriculture for cropland, only two to four times a year harvesting, and optimal growth conditions that involve energy-consuming techniques for further processing [40]. In contrast, seaweeds do not need freshwater and land for their cultivation and can grow in salt water or waste water [40]. They have high photosynthetic efficiency and are fast-growing organisms with biomass yields that are significantly higher than those for terrestrial plants [40,41]. Moreover, macroalgae have low lignin content and consequently, are suitable for the enzymatic downstream processing with milder, less energy consuming and less costly pretreatments compared to plant biomass [42]. Since almost one-third of all known marine fungal species is associated with algae [43], their enzymes are likely well adapted to degrade the polymers in macroalgal cell walls.

In the present study, we isolated and identified marine fungi from the brown seaweed *Fucus* sp. to investigate their abilities to degrade algal polymers. Isolated seaweed derived fungi were analyzed for the production of algal and plant biomass degrading enzymes to expand the existing knowledge of aquatic carbon utilization by marine fungi and the potential of marine fungal enzymes in various industrial applications.

## 2. Materials and Methods

### 2.1. Materials and Culture Conditions

*Fucus* sp. was used to isolate fungi by sampling them with a small brush and then plating this onto malt extract agar (MEA) plates with seawater, followed by incubation at 25 °C for 2–5 days. For sporulating fungi, axenic cultures were derived by streaking a small amount of conidia, collected with the tip of an inoculation needle, on MEA plates, which allowed conidia to separate. After 24 h to 48 h of incubation, plates were observed under a dissection microscope at 50× magnification and single germinating conidia were collected and transferred to MEA and incubated at 25 °C for one week to obtain pure cultures. For non-sporulating fungi, pure cultures were obtained by transferring actively growing hyphal tips onto fresh MEA plate.

The brown seaweed, *F*. *vesiculosus*, was collected in May 2013 from the beach at Scheveningen, the Netherlands. After collection, the samples were washed extensively with water to remove sand, salt and other contaminants. The material was then dried at 60 °C and ground in a Retch mill to particles with a size ≤20 µm. The same method was used for other seaweeds (*Fucus* sp., *L*. *digitata*, *Saccharina* sp., *Sargassum* sp., *U*. *lactuca*) that were collected at NIOZ (Texel, The Netherlands) from either incubation tanks with seawater or directly from the sea. The ground biomass was then used as substrate for the growth profile of the fungi. 

### 2.2. Growth and Utilization of Algal Carbon Sources

The seaweed isolates, the terrestrial fungus *Aspergillus nidulans* FGSC A4 [44], and *Aspergillus sydowii* CBS 593.65, a pathogen of corrals, that has both a terrestrial and a marine life style [45], were grown on MEA to produce spores, which were harvested with ACES buffer [10 mM N-(2-acetamido)-2-aminoethanesulfonic acid] (Sigma-Aldrich, Zwijndrecht, The Netherlands), 0.02% Tween 80, pH 6.1–7.5]. The two Aspergilli were chosen as reference strains as they enable comparison to a terrestrial species (*A. nidulans*) and a species that lives both in terrestrial and marine biotopes (*A. sydowii*). Growth profile of these isolates was performed in duplicate using aspergillus minimal medium (AMM) [46] with 1% (*w*/*v*) glucose or 1% (*w*/*v*) powdered brown seaweed, *F. vesiculosus*. Agar plates were inoculated in the center with 10^6^ spores mL^−1^ or with an agar plug and incubated at 25 °C for 3 days.

Nine selected isolates, and two reference strains, *A. nidulans* and *A. sydowii*, were grown in duplicate on AMM with 1% (*w*/*v*) different macroalgae species, or 1% (*w*/*v*) wheat bran, or 1% (*w*/*v*) glucose, or 1% (*w*/*v*) agar at 25 °C for 3–4 days.

For liquid cultures, 50 mL of AMM containing different carbon sources in a 250 mL flask was inoculated with 10^6^ spores mL^−1^ and incubated at 25 °C, 200 rpm. Tested carbon sources were 1% (*w*/*v*) alfalfa meal, 1% (*w*/*v*) corn powder, 1% *U. lactuca,* and 1% *L. digitata*.

### 2.3. DNA Extraction, PCR Amplification and Sequencing

For molecular analysis, fungi were grown on MEA covered with a polycarbonate membrane containing 0.1 µm pores (GVS Life Sciences, Sanford, ME, USA) at 25 °C for 4 days. Fresh mycelium removed from the polycarbonate membrane with a sterile loop and DNA was extracted using the Wizard^®^ Genomic DNA Purification Kit (Promega, Leiden, The Netherlands) according to the manufacturer’s instructions. Parts of the following loci were amplified and sequenced for all isolated fungi: internal transcribed spacer (ITS) regions and intervening 5.8S nuclear ribosomal RNA (nrRNA) gene; large subunit (LSU), partial nrRNA gene large subunit (28S). The genera *Cladosporium*, *Penicillium*, *Clonostachys, Rhizopus, Epicoccum, Trichoderma, Aspergillus, Alternaria*, *Engyodontium*, *Exophiala*, *Symmetrospora*, *Cryptococcus*, and *Leucosporidium* were identified based on the sequencing results of the ITS and LSU regions. The predominant genera were *Cladosporium* (five isolates) and *Penicillium* (four isolates). Four yeast isolates were identified and represented the genera *Exophiala*, *Symmetrospora*, *Cryptococcus*, and *Leucosporidium*. 

For the selected nine isolates, several additional loci were amplified and sequenced: ACT, partial actin gene; CMD, partial calmodulin gene; TEF, parts of the elongation factor 1α; BTUB, partial β-tubulin fragments. 

The primers V9G [47] and LR5 [48] /RLR3R [49] were used to amplify the ITS + LSU region and primers Bt2a and Bt2b [50] were used to amplify BTUB by using the following PCR program: initial denaturation at 94 °C for 5 min, followed by 35 cycles of 94 °C for 30 s, 52 °C for 30 s, 72 °C for 2 min, and finally an additional 7 min at 72 °C. The primers ACT-512F and ACT-783R were used for ACT [51], primers CMD5 and CMD6 for CMD [52] and primers EF1c and EF6 for TEF [53]. The following PCR program was used: the initial denaturation step was done at 94 °C for 5 min, followed by 40 cycles of denaturation at 94 °C for 30 s, annealing at 52 °C for 30 s, and elongation at 72 °C for 30 s [54]. 

All PCR products were purified using Wizard^®^ SV Gel and PCR Clean-Up System (Promega, USA). Purified PCR amplicons were sequenced by the Macrogen Europe, the Netherlands, using the same primer pairs used for PCR amplification, with additional sequence reactions set up for ITS with ITS4 and ITS5 primers [55] and LSU with primers NL1 and LR5 [48]/RLR3R [49].

Contigs were assembled by using the forward and reverse sequences with the program SeqMan from the LaserGene v. 9 package (DNAstar, Madison, WI, USA). Each sequence was subjected to BLAST search (https://blast.ncbi.nlm.nih.gov/Blast.cgi) and to Pairwise sequence alignment (http://www.westerdijkinstitute.nl/Collections/) to verify its identity. All the sequences generated in this study were deposited in GenBank. The nine selected fungal isolates were deposited in the culture collection (CBS) of the Westerdijk Fungal Biodiversity Institute, Utrecht, The Netherlands.

### 2.4. Enzyme Assays

Culture samples (3 mL) from shaken cultures were taken at 60 h and 120 h, and centrifuged for 10 min, at ~10,000× *g*, 4 °C to separate the solid fraction from the supernatant. The supernatants from duplicate flask cultures were used for enzymatic assays. Endoglucanase and xylanase activities were determined against carboxymethyl cellulose (CMC) and beechwood xylan (Sigma–Aldrich, Zwijndrecht, Netherlands), respectively. The assays contained a total volume of 100 µL using 10–50 µL of culture filtrates and 50 µL of 1% substrate in 100 mM sodium acetate buffer pH 5.0. The samples were incubated in microtiter plates for 30 min at 50 °C. Subsequently, 100 µL of supernatant was mixed with 100 µL 3, 5-dinitrosalicylic acid (DNS) solution. After an incubation of 30 min at 95 °C, absorbance was measured at 540 nm in a microtiter plate reader (FLUOstar OPTIMA, BMG LabTech, Ortenberg, Germany). 

Alginase, carrageenase, fucoidanase and laminarinase activities were assayed using sodium alginate, carrageenan, fucoidan from *F. vesiculosus* and laminarin from *L. digitata*, respectively (Sigma–Aldrich, Germany). The assay contained a total volume of 100 µL using 10–50 µL of culture filtrates and 50 µL of 0.5% substrate in 100 mM sodium acetate buffer pH 5.0. The samples were incubated in microtiter plates for 60 min at 50 °C. Reactions were stopped by addition of 100 µL DNS solution.

Agarase activity was estimated using agar and agarose (Sigma–Aldrich, Germany) as substrates. The assay contained a total volume of 100 µL using 10–50 µL of culture filtrates and 50 µL of 0.25% substrate in 100 mM sodium acetate buffer pH 5.0. The samples were incubated in microtiter plates for 24 h at 50 °C. Reactions were stopped by addition of 100 µL DNS solution. 

Culture filtrates were also assayed using *p*-nitrophenol-linked substrates (4-nitrophenyl β-d-xylopyranoside, 4-nitrophenyl β-d-glucopyranoside, 4-nitrophenyl α-l-fucopyranoside, potassium nitrophenyl sulphate; Sigma-Aldrich). The assays contained a total volume of 100 µL using 10–50 µL of the sample and 50 µL of 2 mM *p*-nitrophenol-linked substrates in 100 mM sodium acetate buffer pH 5.0. Samples were incubated in microtiter plates for 30 min at 50 °C. Reactions were stopped by addition of 100 µL 0.5 M Na_2_CO_3_. Absorbance was measured at 405 nm in a microtiter plate reader. 

The activities were calculated using a standard curve ranging from 0 to 2 g·L^−1^ glucose or ranging from 0 to 20 nM *p*-nitrophenol per assay volume for *p*-nitrophenol-linked substrates. Enzymatic activities results were expressed in U of enzymatic activity where 1 U corresponds to the amount of enzyme necessary to release 1 μmol of equivalent reducing sugar or nitrophenol per minute of reaction. The data obtained are the results of two independent biological replicates and for each replicate three technical replicates were assayed.

## 3. Results

### 3.1. Identification of the Fungal Isolates from Fucus sp.

The twenty-two isolates that were obtained from *Fucus* sp. represented nine genera of the Ascomycota, three genera of the Basidiomycota and one genus of the Mucoromycota. 

The taxonomic information given by the ITS and LSU sequences was insufficient to identify species level of the isolates with exception for *Rhizopus oryzae* (Table 1). Therefore, strains that had the best growth on algal biomass compared to glucose and wheat bran were further sequenced for species identification. *Cladosporium sphaerospermum*, *Cladosporium ramotenellum*, *Cladosporium europaeum*, and *Epicoccum nigrum* were identified based on TEF and ACT sequences, while *Clonostachys rosea* was identified based on BTUB (Table 1). *Penicillium brevicompactum* was identified using BTUB and CMD sequences, and *Trichoderma paraviridescens* was identified using only TEF (Table 1).

### 3.2. Growth and Utilization of Algal Carbon Sources

**Figure 1:**
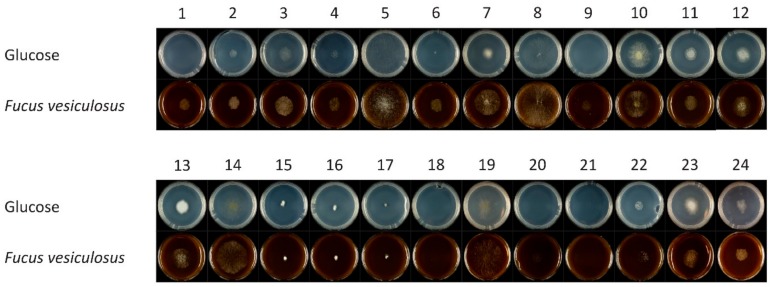
Growth of fungal strains isolated from brown seaweed *Fucus* sp. on glucose and brown seaweed, *F. vesiculosus*. 1: *C*. *ramotenellum*; 2: *P*. *brevicompactum*; 3: *C*. *rosea*; 4: *C*. *europaeum*; 5: *R*. *oryzae*; 6: *C*. *ramotenellum*; 7: *E*. *nigrum*; 8: *T*. *paraviridescens*; 9: *C*. *sphaerospermum*; 10: *Aspergillus* sp.; 11: *Penicillium* sp.; 12: *Penicillium* sp.; 13: *Penicillium* sp.; 14: *Alternaria* sp.; 15: *Engyodontium* sp.; 16: *Engyodontium* sp.; 17: *Exophiala* sp.; 18: *Cladosporium* sp.; 19: *Alternaria* sp.; 20: *Symmetrospora* sp.; 21: *Cryptococcus* sp.; 22: *Leucosporidium* sp.; 23: *Aspergillus nidulans*; 24: *Aspergillus sydowii*.

Fungal strains isolated from the brown seaweed *Fucus sp.* and two reference strains (*A. nidulans* FGSC A4 and *A. sydowii* CBS 593.65) were grown on minimal medium containing 1% of brown macroalgae *F. vesiculosus* and 1% of glucose as a control (Figure 1). Growth profiles showed that *C*. *ramotenellum* (two strains), *P*. *brevicompactum*, *C*. *rosea*, *C*. *europaeum*, *R*. *oryzae*, *E*. *nigrum*, *T*. *paraviridescens*, *C*. *sphaerospermum* grew better and denser on macroalgae than on glucose compared to the rest of the strains. *A. sydowii* CBS 593.65 grew similar on both substrates, while *A. nidulans* FGSC A4 had reduced growth on brown macroalgae (Figure 1). 

**Figure 2:**
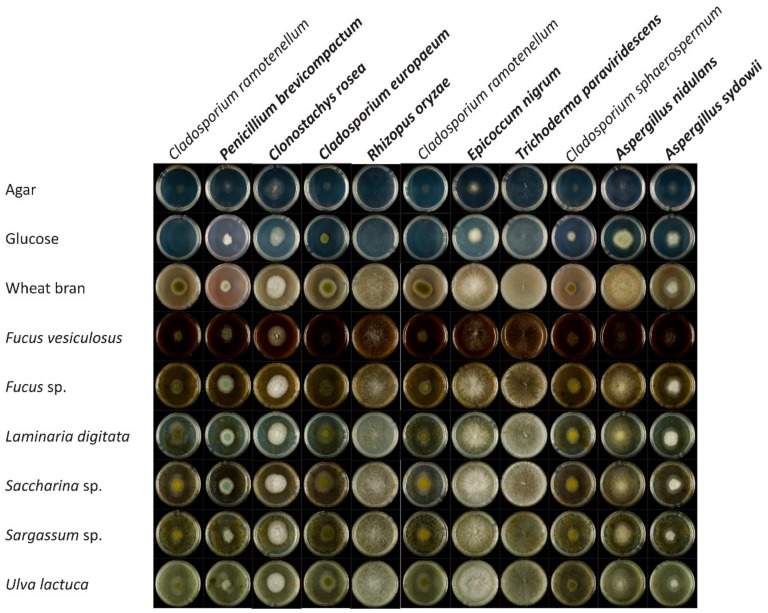
Growth of selected macroalgae derived strains on brown seaweed (*Fucus* sp., *F. vesiculosus*, *L. digitata*, *Saccharina* sp., *Sargassum* sp.) and green seaweed (*U. lactuca*). Strains that were further selected for evaluation of (hemi-)cellulolytic and algal specific enzymatic activities are in bold.

Strains that had better growth on brown seaweed *F. vesiculosus* were selected for more detailed growth analysis together with *A. nidulans* FGSC A4 and *A. sydowii* CBS 593.65. These strains were grown on minimal medium containing 1% brown seaweed (*Fucus* sp., *F. vesiculosus*, *L. digitata*, *Saccharina* sp., *Sargassum* sp.), or 1% green seaweed (*U. lactuca*). Fungal isolates were also grown on 1% wheat bran, 1% glucose, and 1% agar as a control. All selected strains had better growth on algal biomass compared to the growth on glucose and agar. However, they had similar growth on wheat bran and algal biomass (Figure 2). Interestingly, none of the selected fungal strains showed preference for any specific algae, but grew well on all algal substrates. *R*. *oryzae*, *E*. *nigrum,* and *T*. *paraviridescens* showed the best growth on all seaweeds, but grew equally well on wheat bran as a terrestrial strain *A. nidulans* (Figure 2). The preferred substrate for *A. nidulans* was wheat bran, while *A. sydowii* grew well on both plant and algal biomass. Growth profiles of four *Cladosporium* strains (*C*. *europaeum*, *C*. *sphaerospermum* and *C*. *ramotenellum* (two strains)) did not reveal significant differences in growth on seaweed biomass (Figure 2). *C*. *europaeum* was chosen to represent the *Cladosporium* group for growth in liquid media and further enzymatic analysis.

### 3.3. Enzyme Activities during Growth on Seaweed and Plant Biomass Substrates

**Figure 3:**
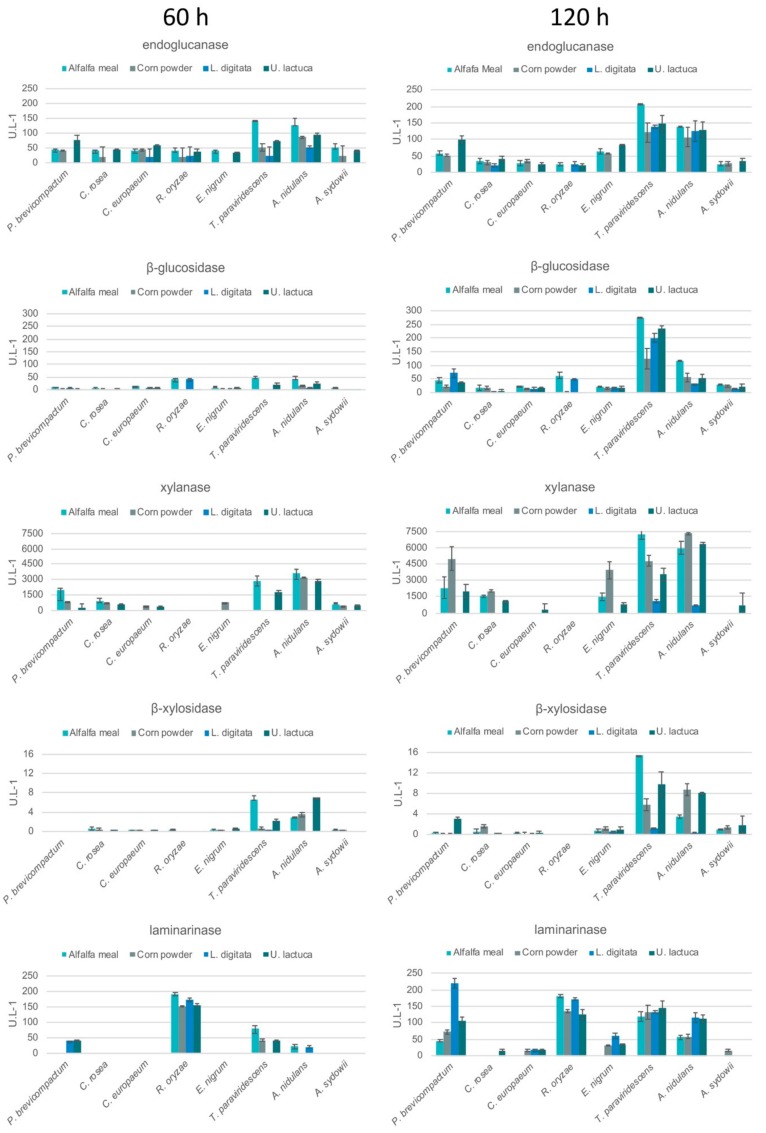

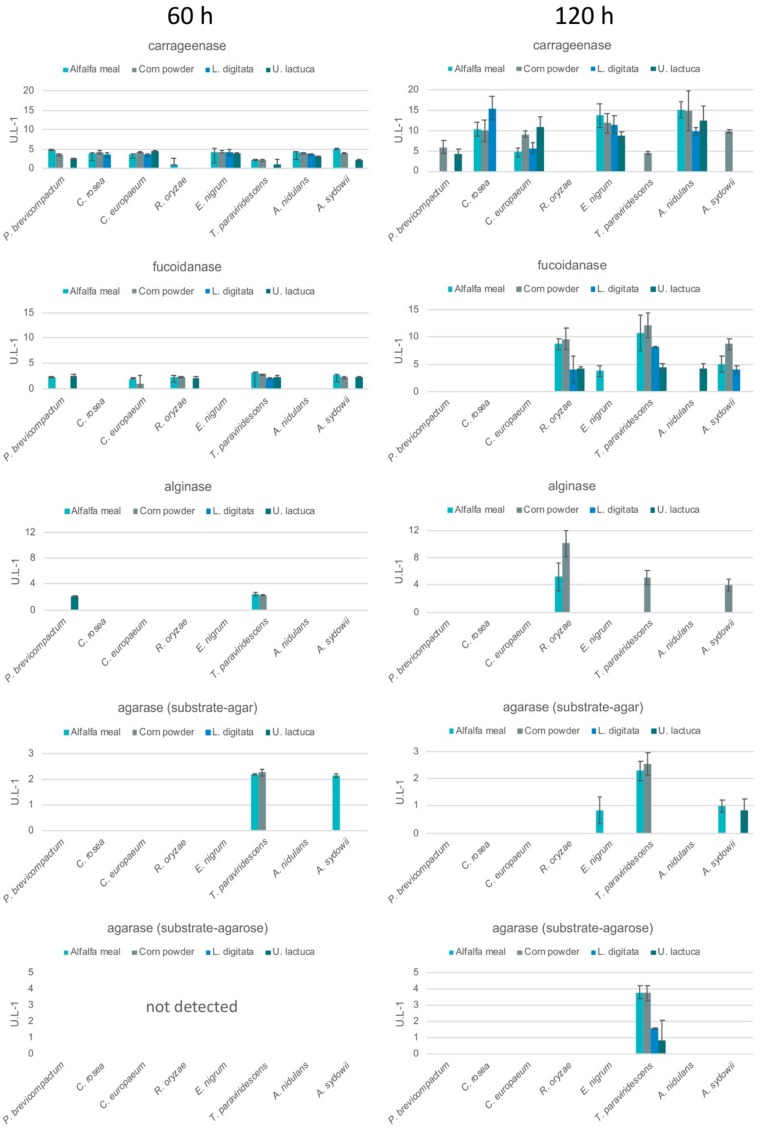
Algal and plant biomass degrading enzyme activities produced by macroalgae derived fungi during growth on seaweed and plant biomass substrates. The error bars indicate the standard deviation of two biological replicate flask cultivations and three technical replicate reactions.

Six strains (*C. europaeum*, *E. nigrum*, *C. rosea*, *P. brevicompactum*, *T*. *paraviridescens,* and *R*. *oryzae*) were evaluated for their (hemi-)cellulolytic and algal specific activities to better understand the abilities of marine fungi to degrade algal and terrestrial biomass. These strains and two reference strains (*A. nidulans* and *A. sydowii*) were grown on minimal media with green algae, *U. lactuca*, brown algae, *L. digitata*, alfalfa meal and corn powder as carbon source. Terrestrial biomass was included to assess whether isolated strains were able to degrade plant material by producing specific enzymes. In general, for all tested species the enzymatic activities increased over time (Figure 3). 

(Hemi-)cellulolytic activities were measured against CMC, beechwood xylan, and *p*-nitrophenol-linked substrates (4-nitrophenyl β-d-xylopyranoside and 4-nitrophenyl β-d-glucopyranoside). A high level of (hemi-)cellulolytic activities was detected during growth on terrestrial (alfalfa meal and corn powder) and algal biomass (green algae, *U. lactuca* and brown algae, *L. digitata*). Enzymes involved in cellulose degradation were detected on corn powder, alfalfa meal, *U. lactuca* and *L. digitata* in most of the species with the exception that there was no endoglucanase activity detected on *L. digitata* in *P. brevicompactum*, *C. europaeum,* and *E. nigrum*. However, β-glucosidase activity was detected in these strains grown on *L. digitata*. Moreover, xylanolytic enzymes were detected mainly on corn powder, alfalfa meal and *U. lactuca*. *R*. *oryzae* did not produce xylanolytic activities, except for β-xylosidases at a very low level after 60 h of cultivation. It has been shown that *R. oryzae* is not able to breakdown xylan significantly as it lacks essential xylanolytic enzymes (xylanases from GH10 or GH11 families), but the *R. oryzae* strain 99–880 genome contains family GH3 or GH43 candidate β-xylosidases [56,57]. However, it has been reported that *R. oryzae* strain NRRL 29086 produced xylanase activities when it was grown on wheat bran [58].

*T*. *paraviridescens* produced the highest endoglucanase and β-glucosidase activities after 120 h of growth on plant and algal biomass. The highest xylanase activity was produced by *E. nigrum*, *P. brevicompactum*, *T*. *paraviridescens* after growth for 120 h on plant and algal biomass. As observed for endoglucanase and β-glucosidase activities, the highest xylanase and β-xylosidase activities were produced by *T*. *paraviridescens*. Although the terrestrial *A. nidulans* produced high levels of (hemi-)cellulolytic activities, the macroalgae isolate *T*. *paraviridescens* produced higher endoglucanase and β-glucosidase activities on all tested substrates, and higher xylanase and β-xylosidase on alfalfa meal after 120 h (Figure 3).

To examine whether isolated marine fungi were capable of degrading seaweed polysaccharides, laminarinase, alginase, carrageenase, fucoidanase, agarase, and sulfatase activities were measured. All fungi were able to produce enzymes involved in algal biomass degradation. However, the production of these activities was lower in comparison with production of cellulases and hemicellulases. Laminarinase (endo-β-1,3-glucanase) activity was detected at the highest levels both on algal and plant biomass. The highest laminarinase activities were produced by *P. brevicompactum*, *T*. *paraviridescens* and *R*. *oryzae*. It has been shown that *Emericellopsis* and *Acremonium* species isolated from the brown seaweed *Fucus* spp. produced laminarinase activities when grown on laminarin [34]. *A. nidulans* was also able to produce laminarinase activities at good levels on all substrates. Furthermore, it produced carrageenase activities at the highest levels on all substrates except for *L. digitata* compared to the isolated from macroalgae strains. In contrast, *A*. *sydowii*, with marine and terrestrial lifestyles, did not produce high levels of enzymatic activities involved in algal or plant polysaccharides degradation. This correlates well with the growth profile of *A*. *sydowii*, as it grew similar well on all tested carbon sources and did not show any outstanding growth.

## 4. Discussion

In the present study, we have explored the macroalgal biomass degrading potential of marine fungi isolated from the brown seaweed *Fucus* sp. The obtained information serves as a source for deciphering the aquatic carbon cycle by marine fungi and exploitation of enzymes involved in seaweed degradation. We have performed isolation and identification of fungal species collected from *Fucus* sp., their ability to grow on different carbohydrates, and correlated the measured enzyme activities to growth on green algae (*U. lactuca*), brown algae (*L. digitate*), alfalfa meal, and corn powder. Our study shows that several macroalgae derived fungi obtained from *Fucus* sp. are able to degrade algal polymers and have furthermore the ability to grow on terrestrial biomass and produce enzymes involved in degradation of plant cell wall polysaccharides.

Analysis of the sequencing results of ITS and LSU regions of fungi isolated from the brown seaweed *Fucus* sp. revealed thirteen different genera: *Cladosporium*, *Penicillium*, *Clonostachys, Rhizopus, Epicoccum, Trichoderma, Aspergillus, Alternaria*, *Engyodontium*, *Exophiala*, *Symmetrospora*, *Cryptococcus*, and *Leucosporidium*. Previous studies have shown similar fungal genera associated with the brown seaweeds *F*. *vesiculosus* (*Aspergillus*, *Coniothyrium*, *Penicillium*, *Alternaria*) [59], *Fucus spiralis* (*Penicillium*), *Fucus serratus* (*Lindra, Lulworthia, Engyodontium, Sigmoidea/ Corollospora complex, Emericellopsis/Acremonium*-like ribotypes [32] and *Aspergillus, Coniothyrium, Penicillium* [59]). *Cladosporium* isolates were dominantly present on the surface of the brown seaweed *Fucus* sp. It was expected as the most frequently isolated genus in marine environment is *Cladosporium* [60]. In addition, all seaweed derived isolates identified in this study are facultative marine fungi and can be found in marine and terrestrial ecosystems. For instance, *C*. *sphaerospermum* [61], *C*. *ramotenellum* [62], *P. brevicompactum* [63], *E. nigrum* [62], *C. rosea* [64], and *R. oryzae* [65] are mainly known from terrestrial environments, but they are also found in deep sea (*C*. *sphaerospermum*) [66], Dead Sea water (*C*. *ramotenellum, P. brevicompactum*) [67], river sediments (*C. rosea*) [68], associated with marine sponges (*E. nigrum*, *P. brevicompactum*) [69,70], bryozoan *Bugula* sp. (*R. oryzae*) [71] or with seagrasses *Posidonia oceanica* (*C. rosea*) [72]. To the best of our knowledge, this is the first study that isolated *T*. *paraviridescens* from a marine environment. However, other species of *Trichoderma* have previously been found in marine sediments, marine sponges, mangroves, brown algae [72,73,74].

The growth profile data showed a clear preference of *C*. *ramotenellum*, *P*. *brevicompactum*, *C*. *rosea*, *C*. *europaeum*, *R*. *oryzae*, *E*. *nigrum*, *T*. *paraviridescens*, *C*. *sphaerospermum* towards the brown seaweed *F. vesiculosus* in comparison with their growth on glucose. These isolates grew well on brown and green algal biomass, indicating no preference for any specific algae. The chemical composition of green and brown algae differs significantly [4,5,6,7], suggesting that isolated species have a broad arsenal of enzymes to degrade algal biomass. Moreover, *Cladosporium* (*C*. *sphaerospermum*, *Cladosporium cladosporioides*) and *Penicillium* (*P*. *brevicompactum*, *Penicillium antarcticum*) species were found associated with both green and brown algae [29,75]. The two reference strains, a terrestrial ascomycete *A. nidulans* and a facultative marine fungus *A. sydowii*, were included in this study to assess physiological differences between isolated marine fungi and well-studied strains with different life styles. *A. nidulans* is a model fungus that is naturally found in the rhizosphere and is commonly used to study the regulation and secretion of lignocellulolytic enzymes [76]. In contrast, *A. sydowii* has both a terrestrial and a marine life style, and produces a broad range of lignocellulolytic enzymes [77]. When assessing growth of the two reference strains on terrestrial and algal biomass, similar growth of *A. sydowii* on wheat bran and green or brown algae and clear preference of *A. nidulans* towards wheat bran were observed. This could be explained by the dual life style of *A. sydowii*, while *A. nidulans* is used to utilizing mainly a terrestrial carbon. Notably, when comparing the growth of terrestrial strain *A. nidulans* on wheat bran with *Fucus* sp derived strains, similar good growth was also observed for *R*. *oryzae*, *E*. *nigrum,* and *T*. *paraviridescens*. These fungi also colonize terrestrial environments, for instance, *R*. *oryzae* was isolated from rice hull [78], *E*. *nigrum* is a litter and leaf decaying fungus [79], and *T*. *paraviridescens* was found on decaying wood [80]. This indicates that these species are widely spread in the terrestrial and marine environment and possibly have a significant role in aquatic and plant biomass degradation. The absence of algal biomass degrading ability in *A. nidulans* together with general terrestrial biomass degradation ability in all tested strains, suggests that terrestrial fungi have lost their ability to degrade marine biomass, or alternatively that the marine isolates have gained their ability to degrade marine biomass upon re-colonizing a marine biotope.

Our analyses examined enzymatic potential of isolated strains grown on corn powder, alfalfa meal, green seaweed *U. lactuca* and brown seaweed *L. digitata*, with focus on the production of plant and algal biomass degrading enzymes. These included enzymes involved in cellulose and xylan (endoglucanase, β-glucosidase, xylanase, and β-xylosidase) and in seaweed polysaccharides (laminarinase, alginase, carrageenase, fucoidanase, agarase, and sulfatase) degradation. The measured enzyme activities were generally in good agreement with the composition of tested substrates confirming that enzymes involved in the degradation of specific polysaccharides would therefore be expected in the culture filtrate of species grown on the substrates consisted of these polysaccharides. Corn powder and alfalfa meal mainly consist of cellulose, xylan, starch, arabinan, and lignin with cellulose and xylan representing the major part of the cell wall [81,82]. In contrast to terrestrial biomass, green seaweed *U. lactuca* contains four polysaccharide families: two major ones, ulvan and cellulose, and two minor ones, xyloglucan and a glucuronan [5], while brown seaweed *L. digitata* carbohydrate components include cellulose, laminarin, alginate, and mannitol [4,12]. Most of the strains were able to produce cellulose degrading enzymes on corn powder, alfalfa meal, *U. lactuca* and *L. digitata*, while xylan degrading enzymatic activities were detected mainly on corn powder, alfalfa meal, and *U. lactuca*. It is not surprising that seaweed derived *T*. *paraviridescens* had the highest (hemi-)cellulolytic activities, as several *Trichoderma* species are known to be good producers of (hemi-)cellulolytic enzymes [83,84].

Based on seaweed polysaccharides degrading enzyme activities measurements, it seemed that there was no correlation between the production of seaweed specific enzymes and the carbon sources tested. Moreover, the marine isolated fungi showed an average capacity to secrete enzymes for the degradation of algal biomass, although these isolates had good growth on brown and green algal biomass. This could be explained by their ability to produce high cellulases and xylanase as these polysaccharides are also important components of the algal cell wall. Possibly, lower levels of enzymes targeting the algal specific polysaccharides already facilitate access to the more common polymers. However, it could also demonstrate our poor understanding of the enzymes involved in algal biomass degradation, as only a small number of enzymes have been identified in contrast to the extensive set that is known for plant biomass. The data obtained here show the existence of different enzymatic approaches to degrade algal biomass. Future cultivation of marine fungi on purified algal polysaccharides such as laminarin, alginate, and carrageenan could give better insights into the mechanisms of utilization of algal carbon by marine derived fungi.

## Figures and Tables

**Table 1:** Marine fungal isolates collected in this study.

Species	Collection No. ^1^	GenBank Accession No.					
		ITS	LSU	ACT	CMD	TEF	Btub
***Cladosporium ramotenellum***	CBS 143784 FP-027-A6	MH102075	MH102097	MH102066		MH102119	
***Penicillium brevicompactum***	CBS 143785 FP-027-A8	MH102076	MH102098		MH102074		MH102072
***Clonostachys rosea***	CBS 143783 FP-027-A3	MH102077	MH102099	MH102067		MH102120	MH102073
***Cladosporium europaeum***	CBS 143786 FP-027-A9	MH102078	MH102100	MH102068		MH102121	
***Rhizopus oryzae***	CBS 143788 FP-027-B4	MH102079	MH102101				
***Cladosporium ramotenellum***	CBS 143787 FP-027-B3	MH102080	MH102102	MH102069			
***Epicoccum nigrum***	CBS 143789 FP-027-C2	MH102081	MH102103	MH102070		MH102122	
***Trichoderma paraviridescens***	CBS 143790 FP-027-C5	MH102082	MH102104			MH102123	
***Cladosporium sphaerospermum***	CBS 143782 FP-027-A1	MH102083	MH102105	MH102071		MH102124	
***Aspergillus* sp.**	FP-027-B1	MH102084	MH102106				
***Penicillium* sp.**	FP-027-A5	MH102085	MH102107				
***Penicillium* sp.**	FP-027-A4	MH102086	MH102108				
***Penicillium* sp.**	FP-027-A7	MH102087	MH102109				
***Alternaria* sp.**	FP-027-B2	MH102088	MH102110				
***Engyodontium* sp.**	FP-027-C4	MH102089	MH102111				
***Engyodontium* sp.**	FP-027-B9	MH102090	MH102112				
***Exophiala* sp.**	FP-027-C6	MH102091	MH102113				
***Cladosporium* sp.**	FP-027-A2	MH102092	MH102114				
***Alternaria* sp.**	FP-027-D2	MH102093	MH102115				
***Symmetrospora* sp.**	FP-027-B6	MH102094	MH102116				
***Cryptococcus* sp.**	FP-027-B7	MH102095	MH102117				
***Leucosporidium* sp.**	FP-027-B5	MH102096	MH102118				

^1^ CBS: culture collection of Westerdijk Fungal Biodiversity Institute, Utrecht, The Netherlands; FP: collection of Fugal Physiology group of Westerdijk Fungal Biodiversity Institute, Utrecht, The Netherlands.
